# Efficacy and clinical outcome of the port-a-cath in children: a tertiary care-center experience

**DOI:** 10.1186/s12957-020-01912-w

**Published:** 2020-06-19

**Authors:** Osama Bawazir, Elaf Banoon

**Affiliations:** 1 Department of Surgery, Faculty of Medicine, Umm Al-Qura University, P.O.box 715, Makkah, 21955 Saudi Arabia; 2 Department of Surgery, King Faisal Specialist Hospital & Research Centre, MBC: j-40, P.O.box 40047, Jeddah, 21499 Saudi Arabia; 3 MBBS, Faculty of Medicine, Umm Al-Qura University, P.O.box 715, Makkah, 21955 Saudi Arabia

**Keywords:** Port-a-cath, Open technique, Percutaneous technique

## Abstract

**Background:**

Implanted vascular access devices play an essential role in the management of pediatric patients. The objectives of this study were to assess our experience with port-a-cath insertion in pediatric patients, report its complications, and compare open versus percutaneous approaches.

**Methods:**

We performed a retrospective cohort study, including 568 patients who underwent port-a-cath insertion between 2013 and 2019 in our center. We grouped the patients according to the technique of insertion into two groups: group 1 (*n* = 168) included patients who had the open approach and group 2 (*n* = 404) included patients who had the percutaneous technique. (*p* < 0.001).

**Results:**

Patients in group 1 were younger (4.10 ± 3.45 years) compared to patients in group 2 (5.47 ± 3.85 years). The main indications of insertion were hematological malignancy 57.74% (*n* = 328), solid organ malignancy 25.18% (*n* = 143), pure hematological diseases 5.46% (*n* = 31), metabolic diseases 2.64% (*n* = 15), and others for poor vascular access 8.8% (*n* = 50). The most common site for insertion in group 1 was the left external jugular (*n* = 136; 82.98%) and the left subclavian in group 2 (*n* = 203; 50.25%). Two hundred and two patients had a central line before catheter insertion (36.6%). Complications during insertion were comparable between both groups (*p* = 0.427). The catheter got stuck in 6 patients; all required additional incision and two needed venotomy. The most common reason to remove the catheter was the completion of the treatment (63.69% and 61.14%, in groups 1 and 2, respectively). The duration of the catheter was comparable between the two groups (13.14 ± 14.76 vs. 14.44 ± 14.04 months in group 1 vs.2; *p* = 0.327).

**Conclusions:**

Open and percutaneous port-a-cath insertions are safe in children with chronic diseases. Port-a-cath improved patients’ management, and complications are infrequent. The most common complications are infection and catheter malfunction, which can be managed without catheter removal in some patients.

## Introduction

Implanted vascular access devices (IVADs) are long-term central venous catheters, which are essential for the management of pediatric patients with chronic disease [1–3]. IVADs have the advantages of avoiding the repeated peripheral venous puncture, less risk of catheter-related infection, and less interference with the activities of the patient [4, 5] Since the introduction of IVADs into clinical practice, they have changed the quality of life of cancer patients [6]. There are different types of access devices available to meet the variability of diagnosis, access requirements, and patient age [7].

Venous access in pediatric cancer patients is a challenging issue. Many chemotherapeutic agents are an irritant to veins and may cause phlebitis. Additionally, these agents may cause ulceration if extravasation occurs. Therefore, oncologists use port-a-catheter frequently to administer chemotherapeutic agents because of their ease of access and low rates of extravasation and infection [8].

On the other hand, the insertion of port-a-catheter and its subsequent care are associated with early and late complications. Complications can be broadly classified into infectious events, venous thrombosis, and mechanical events [9]. Some rare complications were reported, such as rupture, embolization, cardiac tamponade, and difficulty in removal [10]. Complications can be divided according to the timing into early complications that occur within 24 h of insertion and late complications occurring after 24 h, which include infection, thrombotic, and embolic complications [11].

There is a limited number of researches on the complications of long-term access devices in our region. The objectives of this study were to assess our experience with port-a-cath insertion in pediatric patients, report its complications, and compare open versus percutaneous approaches.

## Patients and methods

### Design and patients

This retrospective observation included a total of 568 consecutive patients who underwent port-a-cath insertion in our institute between 2013 and 2019. We collected information regarding patient’s MRN, age, gender, primary diagnosis, the type of devices used, location of insertion, date of insertion and removal, indication for insertion, complications, duration of the catheter, the need for a central line before insertion, and number of the port-a-caths inserted from electronic patient medical records. We excluded patients who had central line insertion or other types of IVADs insertion.

### Ethical considerations

The Research Ethics Committee of King Faisal Specialist Hospital and Research Center, Jeddah, Saudi Arabia, approved the study protocol and data collection for this study. The committee waived patients’ consent because of the retrospective nature of the study. (IRB2019-68; Ref: SURG-J/29/41).

### Surgical technique

Pediatric surgeons implanted all the devices under general anesthesia. All patients received antibiotic prophylaxis before insertion. We flushed the catheter with 3–6 mL of heparinized normal saline (100 IU/mL) at the time of insertion. Two techniques were used for catheter insertion: open cut-down technique (group 1, *n* = 168) and percutaneous technique (group 2; *n* = 404).

### Statistical analysis

We performed statistical analysis using Stata 16 (Stata Corp, College Station, TX, USA). We presented the continuous variables as mean and standard deviation and categorical variables as number and percent. Continuous variables were compared with two-sample independent *t* test and categorical variables with chi-square or Fisher’s exact test if the expected frequency is less than 5. A *p* value of less than 0.05 was considered statistically significant.

## Results

A total of 721 port-a-catheters were implanted in 568 patients during the study period. Patients who had open insertion were younger compared to patients who had percutaneous technique. Conditions requiring port-a-cath insertion were hematological malignancy 57.74% (*n* = 328) (acute lymphocytic leukemia (*n* = 231, 40.9%), lymphoma (*n* = 63; 11.2%), acute myeloid leukemia (*n* = 19; 3.4%), other hematological malignancies (*n* = 21; 3.7%)), solid organ malignancy 25.18% (*n* = 143), pure hematological diseases 5.46% (*n* = 31), metabolic diseases 2.64% (*n* = 15), and others for poor vascular access 8.8% (*n* = 50) (Table 1)

**Table 1:** Preoperative data (continuous variables were presented as mean and standard deviation and categorical variables as number and percent)

	Group 1 (*n* = 164)	Group 2 (*n* = 404)	*t*/chi-square	*p* value
Age (years)	4.1 ± 3.5	5.5 ± 3.9	− 3.95	< 0.001
Male	103 (62.8%)	242 (59.9%)	0.41	0.52
Diagnosis			10.19	0.04
Hematological malignancy	88 (53.7%)	241 (59.7%)		
Solid organ malignancy	49 (29.9%)	94 (23.2%)		
Pure hematological disease	4 (2.4%)	27 (6.7%)		
Metabolic disease	3 (1.8%)	12 (3%)		
Others for poor vascular	20 (12.2%)	30 (7.4%)		
Access				
Reason of insertion			2.84	0.25
Chemotherapy	141 (86%)	357 (88.4%)		
Blood product	1 (0.6%)	0		
Supplements	22 (13.4%)	47 (11.6%)		
Location of insertion			568	<0.001
Left external jugular	136 (82.9%)	0		
Right external jugular	28 (17.1%)	0		
Left subclavian	0	203 (50.3%)		
Right subclavian	0	65 (16.1%)		
Left internal jugular	0	112 (27.7%)		
Right internal jugular	0	22 (5.5%)		
Other	0	2 (0.5%)		
Need central line before insertion	59 (36%)	143 (35.4%)	0.017	0.9

The most common site for insertion in group 1 was the left external jugular (*n* = 136; 82.98%) and the left subclavian in group 2 (*n* = 203; 50.25%). Two hundred and two patients had central line before catheter insertion (36.6%).

**Fig. 1:**
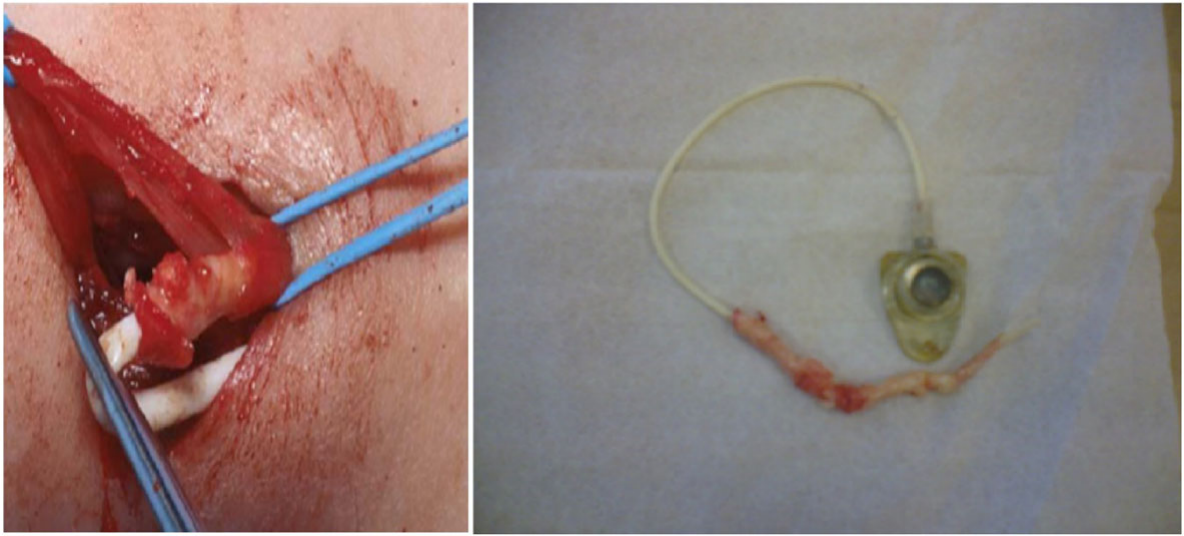
Operative view of a stuck catheter requiring venotomy for removal with evident calcification

**Fig. 2:**
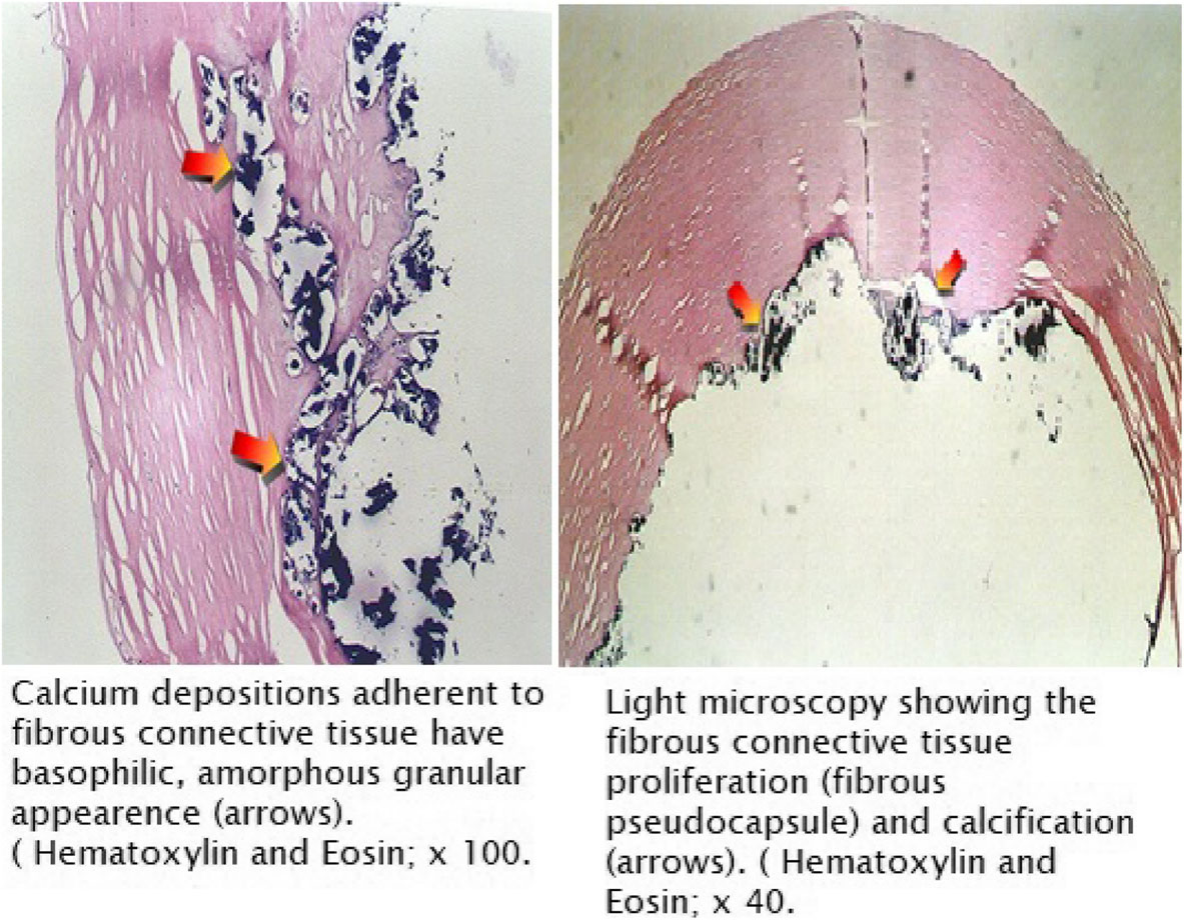
The microscopic examination of removed stuck catheter showing the extensive calcification on the catheter

**Fig. 3:**
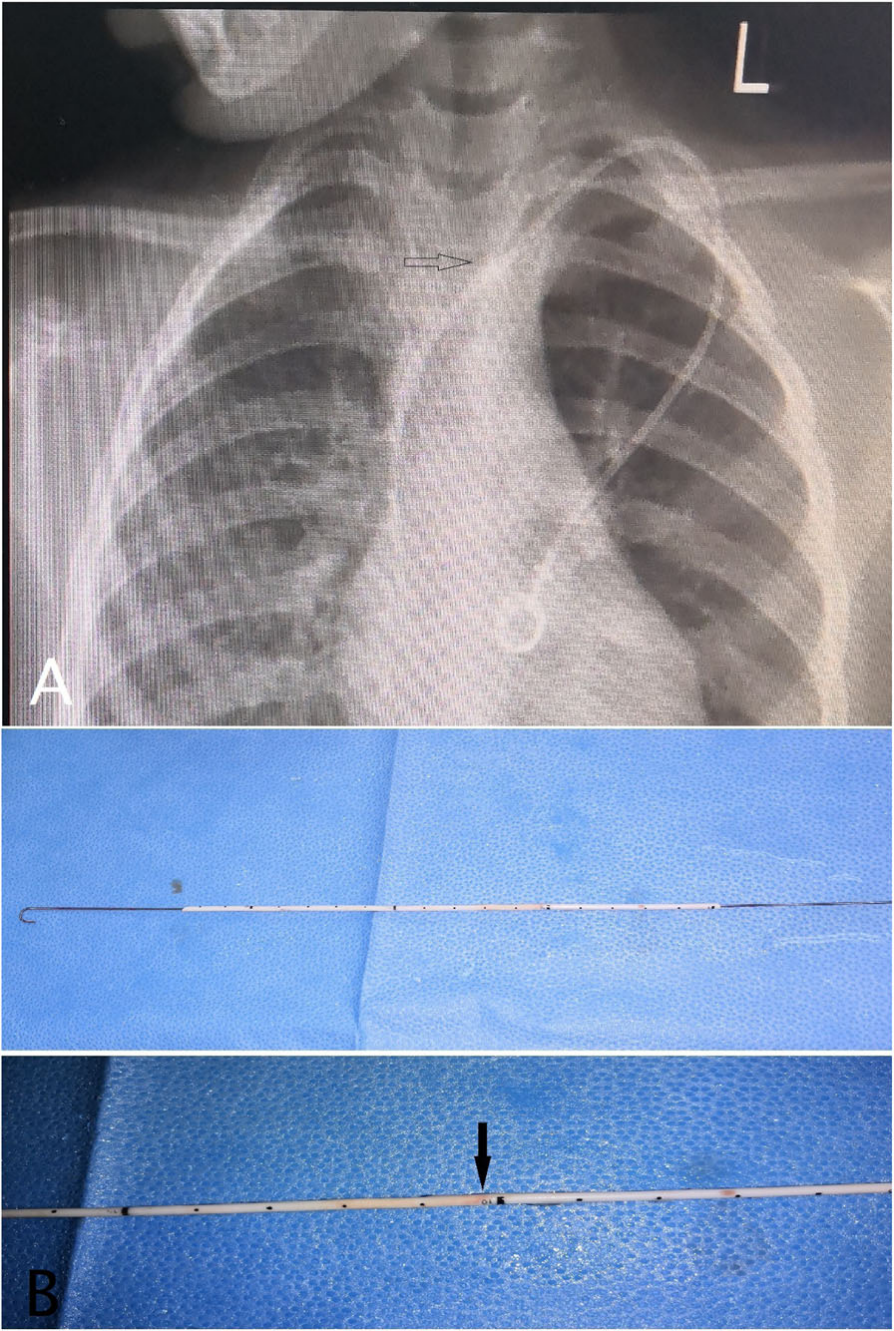
**a** Chest X-ray showing thickening of the catheter at the subclavian vein (black arrow).The catheter was stuck and removed by inserting a good-sized guidewire in it and pull it. **b** The catheter after removal. The black arrow shows the site of the adhesion on the catheter

**Fig. 4:**
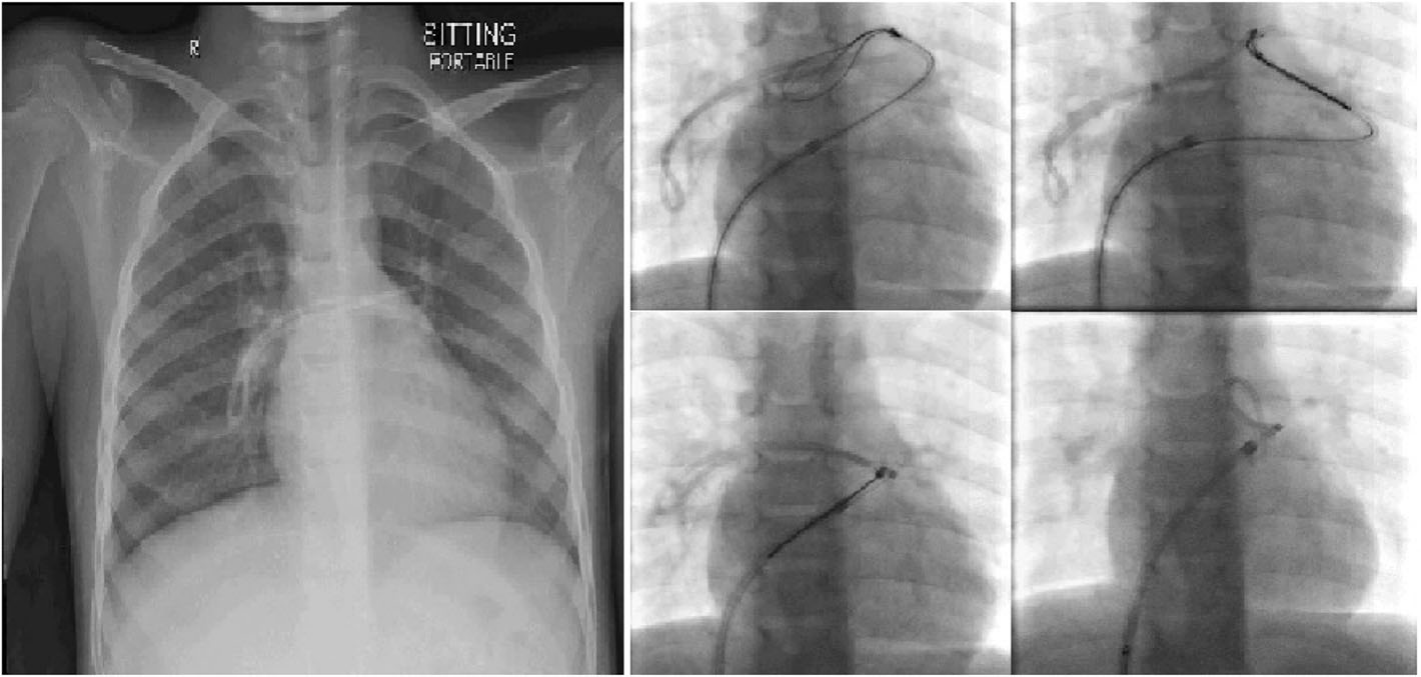
Chest X-ray and fluoroscopy showing a dislodged catheter which was retrieved by the interventional radiologists

**Fig. 5:**
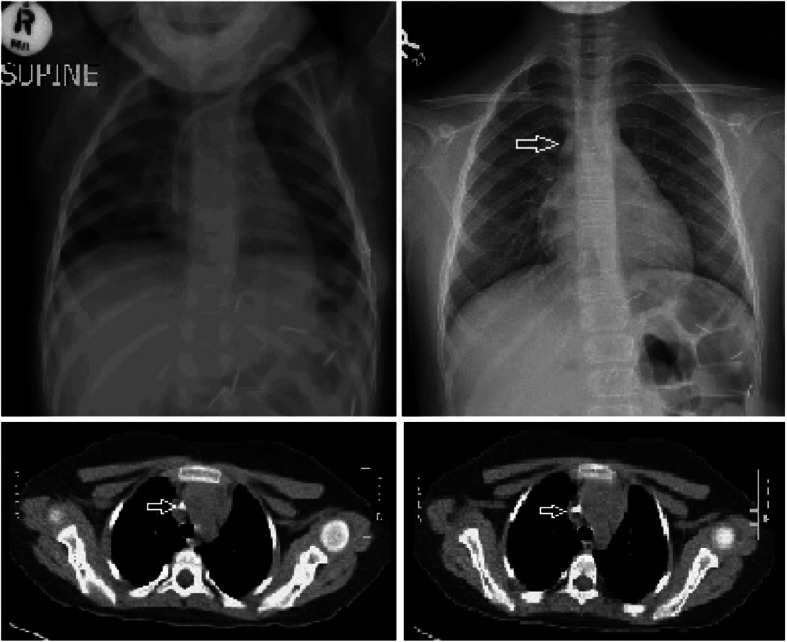
Follow-up chest X-ray and chest CT scan after 4 years of catheter removal showing the retained part of the catheter with no thrombosis of the superior vena cava

During removal, the catheter got stuck in 6 patients; all required additional incision and two needed venotomy (Figs. 1 and 2). Three were removed by inserting guidewire in the catheter and traction (Fig. 3), and one required a combined approach with interventional radiology (Fig. 4). Longer duration of the catheter was significantly associated with stuck complications, and all stuck catheters occurred in patients who had catheters for more than 2 years. Four cases had dislodgement of a catheter in the right atrium and right pulmonary artery and were discovered during removal or with extravasation. We reported one patient with embed distal part of the catheter in the superior vena cava, and it was discovered later after the removal of the port-a-cath. We managed this patient conservatively and left the catheter for the last 4 years without any side effects (Fig. 5).

The most common reason to remove the catheter was the completion of the treatment. The most common complication which required catheter removal was infections followed by malfunction and thrombosis, leakage, catheter break, and hematoma. The duration of the catheter was comparable between the two groups (Table 2).

**Table 2:** Operative and postoperative outcomes (continuous variables were presented as mean and standard deviation and categorical variables as number and percent) (*PE* pulmonary embolism)

	Group 1 (*n* = 164)	Group 2 (*n* = 404)	*t*/chi square	*p* value
Complication during insertion			4.94	0.43
Hematoma	0	4 (1%)		
Multiple site insertion	5 (3.1%)	15 (3.7%)		
Conversion to cut down	3 (1.8%)	15 (3.7%)		
pneumothorax/hemothorax	0	4 (1%)		
Complication during removal			0.86	0.87
Stuck	2 (1.2%)	4 (1%)		
Need for another incision/venotomy	5 (3.1%)	9 (1.2%)		
Bleeding	1 (0.6%)	1 (0.3%)		
Migration to heart	1 (0.6%)	3 (0.7%)		
Reason to remove the catheter			7.49	0.38
Complete treatment	107 (63.7%)	247 (61.1%)		
Infection	19 (11.6%)	75 (18.6%)		
Malfunction/thrombosis/blockage	14 (8.5%)	27 (6.7%)		
Leakage	0	5 (1.2%)		
Catheter break with or without PE	2 (1.2%)	5 (1.2%)		
Bone marrow transplantation	18 (11%)	38 (9.4%)		
Hematoma	1 (0.6%)	1 (0.3%)		
Death (related to the primary disease)	2 (1.2%)	5 (1.2%)		
Refuse treatment	1 (0.6%)	1 (0.3%)		
Need another catheter insertion	36 (22%)	117 (29%)		0.09
Duration of catheter (months)	13.1 ± 14.8	14.4 ± 14	− 0.98	0.33

## Discussion

Port-a-cath became increasingly popular for the management of cancer patients since their outcome is comparable to the tunneled central lines with low risk of infection [2]. In our facility, we prefer the port-a-cath over the tunnel central line or peripherally inserted central lines as long-term venous access because of the ease of use for families and healthcare professionals.

In this study, we aimed to analyze our experience with the insertion of port-a-cath, report its complications in pediatric patients, and compare both techniques of insertion. Patients with neoplasms, hematologic disorders, and patients that require long-term supplements require long-term venous access, and IVADs helped to improve the quality of care in those patients. Most children with oncological diseases in our facility had port-a-cath insertion as the standard of care

In our study, the majority of the port-a-cath inserted were placed percutaneously in the left subclavian vein because of the ease of implantation. Additionally, it provides long intravascular length, which is very important in small infants because as they grow, the catheter can be pulled out before they finish their treatment. Visualization of the central vein at the time of insertion of the venous catheter is important in reducing the rate of failure and complications relating to damage to adjacent structures. Therefore, we inserted most of the catheters under ultrasound guidance. A study reported that the port-a-cath implantation method without guidance was less effective than ultrasound-guided [12].

The average duration of the catheter in our study was 14 months and is consistent with the published series, which ranged between 12 and 22 months [5]. The use of IVADs may be associated with complications, most of which can be effectively controlled without the removal of the catheter. Insertion and maintenance are important to minimize iatrogenic injuries and reduce complications related to the catheter. Infection was the most common complication in our series, followed by thrombosis. Thrombosis and infection were reported in associated with hematological malignancy, which could be attributed to abnormal immune response and viscosity of the blood, making the central venous catheter susceptible to thrombosis and, subsequently, infections [13].

Prior central line insertions, more than one device insertion, and a long duration of the catheter were risk factors for infection [14]. In our study, 94 (16.5%) catheters were removed because of infection. The education and training programs for the patients and healthcare providers involved in the insertion and care of catheters will help reduce the infection rate. Our study showed that catheters malfunction and thrombosis, which required ports replacements was 7%, which is consistent with what was reported in the literature (5%) [3]. Catheter blockage is suspected when there is a failure in infusion, or the catheter fails to withdraw blood.

Most of the complications have been related to insertion, and scarce data were published about the complications associated with their removal. The detection and treatment of complications related to the extraction of central venous catheters should be emphasized. We had 4 cases who developed dislodgement of a catheter in the right atrium and right pulmonary artery. The causes could be a poor connection to the port, catheter damage at the pinching point below the clavicle, or incorrect catheter position. The dislodgment rate of port-a-catheters reported in the literature was 1.4 to 3.6% [15, 16] with an average of 2.4% [17]. This rate is higher than what was reported in adults, which ranged from 0.3 to 1.5% [13, 18] Most catheters were broken at the site of connection [19]. This could be attributed to the manual assembly of the catheters to the port by the surgeon in the operating room or because of the hyperactivity of patients in this age group. Surprisingly, patients with dislodged catheter did not present with respiratory symptoms and did not require supplementary treatment pre or post removal of the dislodged catheter. However, most dislodgements present with only irrigation resistance or even without symptoms or signs [20].

Long-term use of central venous catheters is known to have peri-catheter adhesions and calcifications. These factors can result in stuck catheters that are difficult to remove. The incidence of stuck catheters is not exactly known, and different results have been reported with an incidence ranged from 0.3 to 2.2% [21]. The long duration of the catheter was the main predisposing factor to this complication [22, 23]. To facilitate removal, a second incision was required in 4 patients and venotomy in 2 patients. Inserting a guidewire into the catheter allows greater traction to be applied to the stuck catheter without fracturing it [24, 25]. Two cases failed this technique, which required removal by venotomy of the left internal jugular vein. Others advocate using the endoluminal dilatation technique to remove stuck port-a-cath [26–28]. We reported one patient with embed distal part of the catheter in the superior vena cava, and it was discovered later after the removal of the port-a-cath.

In our experience, the best management of a retained fragment, if it is free-floating, is to remove it by the help of interventional radiologists or cardiologists. However, if it is fixed to the wall of the major vascular structure, it is wise to leave the catheter in place because removal may lead to fatal complications such as hemorrhage and the need for a major surgery like sternotomy with pulmonary arteriotomy [29]. As soon as the catheter becomes unnecessary, it is crucial to be removed with caution to avoid complications related to its extraction, such as bleeding, infection, air embolism, and catheter embolism. As a safety measure, we try to remove all the ports after 2 years of placement.

In comparing both approaches for port-a-cath insertion, we did not find a significant difference between both techniques in insertion and removal complications. This indicates that both approaches are safe, and the choice of the approach should be tailored according to the patients’ characteristics.

### Limitations of the study

The limitations of our study were the retrospective nature and single-centered study. Additionally, there was loss of data during the study period. However, the study presents a large experience in the management of pediatric patients with port-a-cath.

## Conclusion

Open and percutaneous port-a-cath insertions are safe in children with chronic diseases. Port-a-cath improved patients’ management, and complications are infrequent. The most common complications are infection and catheter malfunction, which can be managed without catheter removal in some patients. It is recommended to remove the catheter before 2 years to avoid complications.

## Data Availability

The datasets generated and/or analyzed during the current study are not publicly available due [patient confidentiality policy in our institution] but are available from the corresponding author on reasonable request.

## Abbreviations

IVADs: Implanted vascular access devices
